# The Possible Correlation between the Patient's Immune Tolerance Level During Cesaerean Section and the Incidence of Subsequent Emergency Peripartum Hysterectomy

**DOI:** 10.1155/2007/63596

**Published:** 2007-11-28

**Authors:** Lukasz Wicherek, Krystna Galazka

**Affiliations:** ^1^Department of Gynecology, Obstetrics and Oncology, Jagiellonian University, 23 Kopernik Street, 31-501 Krakow, Poland; ^2^Department of Patomorphology, Jagiellonian University, 17 Grzegorzecka Street, 31-531 Krakow, Poland

## Abstract

*Introduction*. Cesarean section is an independent risk factor for peripartum hysterectomy. As a method of delivery, cesarean section may interfere with a number of molecular changes that occur at the maternal-fetal interface during the course of labor. *Methods*. The level of CD3, CD56, CD25, and CD69 antigen immunoreactivity was assessed by immunohistochemistry in 26 decidual tissue samples. The tissue samples were obtained from 18 women who underwent cesarean sections at term and from 8 women who underwent cesarean hysterectomies. *Results*. An increase in the activity and infiltration of immune cells in the decidua sampled during the spontaneous beginning of labor was observed. The further progression of labor was accompanied by a decrease in the number and activity of immune cells. The number of CD56+ and CD3+ cells in the decidua was statistically significantly lower in patients who had undergone cesarean hysterectomies than in those who had had cesarean sections at term. *Conclusion*. Abnormal immune response during labor may increase the risk for peripartum hysterectomy.

## 1. INTRODUCTION

Peripartum hysterectomy, a life-saving procedure, is performed more often on women who have undergone cesarean sections (cesarean hysterectomy) than on those who experience normal labor (postpartum-hysterectomy) [1]. Most studies report the rate of occurrence for peripartum hysterectomies as between 0.2 and 1.5 per 1000 deliveries [2–4]. In some studies, however, the incidence is reported to be as high as 2.25 or 2.7 per 1000 deliveries [1, 5]. The chief indications for hysterectomy are abnormal placental detachment and atonic postpartum hemorrhage [1]. In recent years, a significant increase in peripartum hysterectomies has been reported, and this increase would seem to correlate with an increase in the number of cesarean sections performed [2]. Not only does cesarean delivery increase the risk for emergency peripartum hysterectomy [4–7], but in cases of repeat cesarean sections there is an associated increase in the risk of a number of other complications, such as abnormal placentation, placenta previa, or uterine sparring [2, 8, 9].

Elective cesarean section (cesarean section without labor) is performed before the physiological alteration of immune tolerance associated with pregnancy and the beginning of spontaneous labor has occurred. The level of immune tolerance during newborn expulsion at term in cases of elective cesarean section appears to differ from the level found in cases of spontaneous vaginal delivery at term. Molecular changes at the maternal-fetal interface are the pivotal mechanism in the alteration of the immune tolerance phenomenon. These changes lead to an increase in the activity of the maternal and fetal immunological systems [10–14]. During spontaneous vaginal delivery, a decrease in placental factors suppressing maternal immune response, including Fas-L, RCAS1 and HLA-G, has been observed along with a drop in the number of CD4CD25 positive suppressive lymphocytes within the decidua [15–19]. On account of their immunomodulating activity, decidual cells, together with placental and maternal immune cells, also participate in the development of immune tolerance phenomenon [20–26].

In the present study we have aimed to assess the number and activity of immune cells in the decidua using samples collected from patients who have had term cesarean sections and from patients who have had postcesarean hysterectomies.

## 2. MATERIALS AND METHODS

### 2.1. Patients

The decidual tissue samples evaluated in our study were obtained from 26 pregnant women who underwent cesarean sections. 18 of the tissue samples were obtained during cesarean sections at term, and 8 from cesarean hysterectomy specimens. Those patients undergoing cesarean section at term (18 cases) were selected from 650 women undergoing cesarean sections in the Department of Gynecology, Obstetrics and Oncology, Jagiellonian University, Krakow, Poland, in 2005. Patients with multiple pregnancies or existing complications of pregnancy, such as preterm deliveries, hypertension, diabetes mellitus, and cases of fetal demise, were excluded from the present study. For the purpose of this study, the histories of patients who had undergone emergency cesarean hysterectomies between 1999 and 2004 were analyzed and 8 were selected. Women on whom peripartum hysterectomies had been performed due to uterine cancer during pregnancy were also excluded from our analysis. The tissue samples derived from the women undergoing cesarean section at term and obtained during surgical procedures were thereafter immediately fixed in 10% buffered formaldehyde solution and sent to the Pathomorphology Department, Jagiellonian University (Karkow, Poland). The decidual tissue samples from the patients having undergone emergency hysterectomies were obtained from the archive files of the Department of Pathomorphology of the Jagiellonian University. The experienced pathomorphologist (K. Galazka) evaluated the routinely stained (hematoxylin and eosin) slides prepared from paraffin-embedded tissue material and selected the material adequate for further analysis. Chosen paraffin blocks were cut and used for immunohistochemistry.

The patient';s consent was obtained in each case.

### 2.2. Immunohistochemistry

Immunohistochemical analysis was performed in the Pathomorphology Department of the Jagiellonian University. Four-micrometer slides from each case, encompassing the decidua, prepared routinely for immunohistochemistry, were stained to visualize the CD3, CD69, CD25, CD56-positive cells (lymphocytes).

In all instances, immunohistochemistry was performed by applying the Envision method using Dako Autostainer. The following antibodies were applied: CD56 (NCAM; NCL-CD56-504, Novocastra) in dilution 1:100, CD69 (NCL-CD69, Novocastra) in dilution 1:25, CD25 (Interleukin-2 Receptor, NCL-CD25-305, Novocastra) in dilution 1:25, CD3 (NCL-CD3p, rabbit polyclonal antibody, Novocastra) in dilution 1:100, according to the manufacturer';s instructions. Visualization of reaction products was performed using AEC (3-amino-9-ethyl-carbazole) as a chromogen (AEC Substrate Chromogen ready-to-use, DAKO, Denmark) for 10 minutes at room temperature. Sections were counterstained with hematoxylin and mounted in glycergel. The numbers of different lymphocytes in the decidua were evaluated. The number of immune cells in an entire specimen and an average cell number per 1hpf (high power field, objective magnification ×40) were calculated. The following scale was used to evaluate the number of cells semiquantitatively: 0—lack of positive cells; +1—–5 positive cells per 1hpf; +2—6–10 positive cells/1hpf, +3—11–20 positive cells/1hpf; +4—more than 20 positive cells per 1hpf.

### 2.3. Statistical analysis

The distribution of variables in the groups of women studied checked with the use of the Shapiro-Wilk test showed that all of the women were different from normal. Therefore, non-parametric testing was employed. The differences between the groups were determined by the Kruskal-Wallis analysis of variance (ANOVA) test. The Mann-Whitney U test was then used as applicable.

## 3. RESULTS

### 3.1. Clinical comparison of analyzed groups of patients: cesarean section at term and peripartum hysterectomy

Since cesarean section is performed at different times during pregnancy, it seems important to compare the parameters characterizing the course of pregnancy and labor in the respective groups of patients studied (see Table 1).

When compared, the basic difference between the groups was related to the parity of the pregnant women studied. Multiparity is an independent risk factor for emergency hysterectomy [7] that could also be related to the status of immune tolerance during pregnancy. The central question of this study is whether the number and activity of immune cells at the maternal fetal interface correlates with the level of immune tolerance during pregnancy.

### 3.2. Immunohistochemical analysis of the immune cells presence and their activity

CD3 positive cells were identified in all the decidual tissue samples derived from patients who had undergone cesarean sections at term and in 25% of the decidual tissue samples derived from patients having had cesarean peripartum hysterectomies. CD56 positive cells were observed in 60% of the decidual tissue samples derived from those patients having had cesarean sections at term and in 13% of decidua tissue samples derived from patients having had cesarean peripartum hysterectomies. CD69 antigen immunoreactivity was observed in 40% of the tissue samples derived from those having undergone cesarean sections at term and in 38% of the tissue samples derived from those having had cesarean peripartum hysterectomies. CD25 antigen immunoreactivity was weak in the samples derived from the group who had undergone cesarean sections at term; it was not detected in the samples from the group who had had cesarean peripartum hysterectomies (see Table 2).

The changes in the number and activity of immune cells were identified in the decidua and correlated with the progression of labor. The study group of patients who underwent cesareans at term was divided into three subgroups according to the degree of uterine cervical ripening and to the presence of uterine contractions during the surgical procedure. Group A consisted of patients not experiencing uterine contractions and with a closed uterine cervical ostium (i.e., caesarean without labor); group B consisted of patients with irregular uterine contractions or the beginning of regular uterine contractions and cervical dilation between 1 and 3 cm (i.e., caesarean section with symptoms indicating the spontaneous beginning of labor); group C consisted of patients with regular uterine contractions and cervical dilation of more than 3 cm (i.e., “caesarean with advanced labor”). The comparison of the number and activity of immune cells between subgroups is presented in Table 3.

We found a statistically significantly higher number of CD3 positive cells and a higher CD69 antigen immunoreactivity level in the decidua from those on whom cesarean sections were performed after the spontaneous beginning of labor (B) in comparison to what was found in the decidua from those on whom cesarean sections were performed without labor (A), and from those on whom cesarean sections were performed during advanced labor (C) (P, resp., (CD3+: P=.01 and P=.04; CD69: P=.03 in both cases). Although the number of CD56 positive cells increased with the progression of labor, the differences noted were not statistically significant.

### 3.3. Comparison of molecular alterations within decidua in cesarean section at term and in cesarean hysterectomy

Our analysis of the indications for cesarean sections (the number of elective cesarean sections), when taking into consideration such factors as the progression of labor during surgery and the gestational age both in the group of patients with cesarean peripartum hysterectomies and in the group with the cesarean sections at term, did not reveal any significant differences. In the group of patients having had cesarean peripartum hysterectomies, significantly higher parity was observed. Also, the average age of these women was significantly higher (resp., P=.01 and P=.02).

Our study group consisted of patients with cesarean sections followed by peripartum hysterectomies who either presented no symptoms of the spontaneous beginning of the labor or were in its very initial stage. Significantly, no women in advanced labor found themselves in this group. The women selected for the control group were at the same stage of labor as those in the study group (caesarean without labor or caesarean section with symptoms indicating the spontaneous beginning of labor). The number and activity of immune cells in the decidua of the group of cesarean peripartum hysterectomies and of the control group were compared. The results obtained are presented in Table 4.

## 4. DISCUSSION

An increase in the activity and infiltration of immune cells in the decidua sampled during the spontaneous beginning of labor was observed. Moreover, the further progression of labor was accompanied by a decrease in the number and activity of immune cells. The number of CD56 positive and CD3 positive cells in the decidua was statistically significantly lower in the samples from those having had cesarean hysterectomies than in the samples of those having had cesarean sections at term.

Risk factors for emergency peripartum hysterectomy include cesarean delivery, prior cesarean, placenta praevia, placenta acreta, and grand multiparity [3, 7, 27]. What, then, is the possible common denominator present in all these different clinical conditions? What common anomaly causing alterations in immune tolerance during the progression of labor and increasing the risk for emergency hysterectomy can we find? Spontaneous labor at term involves gradual changes in maternal immune tolerance. For example, the infiltration of leukocytes into the upper and lower uterine segments seems to play an important role in the course of normal parturition [13, 28]. A peripartum increase in immune response has already been described [10, 12, 29, 30]. Elective cesarean section is a surgical procedure performed prior to the spontaneous beginning of labor and, therefore, prior to the molecular changes that are responsible for the initiation and beginning of the course of labor. Cesarean section is one of the primary risk factors for peripartum hysterectomy [2, 6, 27, 31]. The most common indication for cesarean section followed by peripartum hysterectomy is malpresentation [27], and this is in itself an indication for an elective cesarean section. In our study, all of the emergency cesarean hysterectomies were performed on patients who had had elective cesareans. The deficit of infiltrating immune cytotoxic cells that is associated with the surgical procedure when performed before the beginning of spontaneous labor may affect the subsequent steps of the immune response. Therefore, elective cesarean performed without the molecular alterations responsible for the progression of labor seems to increase the risk for peripartum hysterectomy.

Abnormal placentation has recently been demonstrated to be a common indication for cesarean section and has been found possibly to lead to peripartum hysterectomy. A previous cesarean increases the risk of abnormal placentation [7, 31]. The verification of placenta percreta in histopathological analysis means the absence of decidua basalis [9, 32–34], which may itself be associated with a low number of immune cells within the decidua. In our study, placenta percreta was identified in 3 patients (37% of cases).

Sindram-Trujillo et al. has observed that the course of spontaneous vaginal delivery is accompanied by an increase in the number of immune cells as well as alterations in their distribution (NK CD56+CD16+ cells and T cell subpopulation) [11, 18]. Bulmer and Lash, however, have observed a decrease in dNK cells in the decidua during the second and third trimesters of pregnancy [35]. Spornitz et al. has suggested that the decrease in the number of these cells may correlate with their function during the first trimester of pregnancy [36] while the increase in NK cells observed by Sindram-Trujillo et al. may be associated with the recruitment of new cells to the decidua. Rukavina and Podack has shown a continuing recruitment of immune cells to the decidua throughout gestation with concomitant phenotype changes in peripheral NK cells [37]. Recently, the recruitment of these cells to the decidua was found to result from the activity of the decidual cells rather than from the physical presence of the embryo [38]. The beginning of the labor correlates strongly with alterations in the pattern of cytokine concentration in the decidua, including IL-8 and IL-6 [39]. Campbell et al. have indicated that the CD56+ cell population derived from multiple lineages can be discriminated by the expression of trafficking molecules. Moreover, CD56+CD16+ cells are extremely sensitive to IL-8 chemotactic activity [24]. In our study, the accumulation of CD56+, CD3+ lymphocytes in the decidua began after the initiation of labor. In patients on whom peripartum hysterectomies were performed, no such increase was observed, which may suggest a correlation between the risk for emergency hysterectomy and the presence of abnormal immune response during labor.

Although the presence of uterine leiomyomas was found to increase the risk of peripartum hemorrhages [40], Sheiner et al. did not observe any correlation between cesarean hysterectomy and these neoplasms. In our study, uterine leiomyomas were observed in 3 cases of peripartum hysterectomy [27].

Women with grand multiparity have been identified as more predisposed to postpartum hemorrhage [41, 42], and grand multiparity is furthermore an independent risk factor for emergency peripartum hysterectomy [4, 7, 9, 27, 33, 43]. In the present study, no nulliparous women were observed in the group on whom emergency hysterectomies were performed. Maymon et al. has shown that grand multiparity is an independent risk factor for peripartum complications such as malpresentation, dysfunctional labor, and massive hemorrhage [43]. Moreover, with each succeeding pregnancy the risk increases, and in the 14th gestation the risk is 3 times higher than in the 7th [43]. Multiparity is related to the repeated development of immune tolerance during pregnancy. Thus, although our samples are insufficient to prove such claims, one may conjecture that with each consecutive labor the risk that the woman may not develop the complex immune response involved in the alteration of immune tolerance level during spontaneous labor increases. It has been shown that great grand multiparity is more frequently associated with the disturbance of uterine cervical ripening and postterm deliveries [42]. Both complications have immunological backgrounds. For instance, Gabrilovac et al. has observed that multigravidas exhibit a significant decline in NK cell activity [44]. This may be a predictor of immune response disturbance in any subsequent pregnancies.

The present study has had certain limitations. First, the small number of patients involved does not allow us to posit that abnormal immune cell activity is a risk factor for emergency hysterectomy. We can only suggest, on the basis of trends, that a probable association exists between an immune response disturbance during labor and the need for emergency hysterectomy. This probable correlation will have to be explored in more detail in further investigations.

## 5. CONCLUSION

Abnormal immune response during labor may increase the risk for peripartum hysterectomy.

## Figures and Tables

**Table 1:** Clinical characteristic of patients.

Variables	Cesarean section at	Emergency cesarean	P value
	term (n=18)	hysterectomy (n=8)	
Maternal age (average ± SD*)	28.6 (±5.7)	38.6 (±7.5)	.0003
Parity (median ± SEM)	1 (±0.20)	4 (±0.64)	.00008
Gestational age (average ± SD)	38.5 (±1.29)	38.12 (±1.35)	.3
Newborn mass (average ± SD)	3031 (±530)	2937 (±425)	.57
Newborn length (average ± SD)	50.11 (±2.69)	52.50 (±3.5)	.33
Apgar score (median ± SEM)	9 (±0.29)	9 (±0.29)	.7

*SD: standard deviation.

**Table 2:** Immunoreactivity of CD3, CD56, CD69, and CD25 antigens within decidua from patients who underwent cesarean section at term and those who underwent an emergency cesarean hysterectomy.

Groups	Antigen	Immunoreactivity				
		*percent (number of cases)				
		0	+1	+2	+3	+4
Cesarean hysterectomy (n=8)	CD3	75 (6)	0 (0)	0 (0)	0 (0)	25 (2)
	CD69	62 (5)	13 (1)	25 (2)	0 (0)	0 (0)
	CD56	87 (7)	13 (1)	0 (0)	0 (0)	0 (0)
	CD25	100 (8)	0 (0)	0 (0)	0 (0)	0 (0)
Cesarean section at term (n=18)	CD3	0 (0)	50 (9)	6 (1)	28 (5)	16 (3)
	CD69	55 (10)	33 (6)	6 (1)	6 (1)	0 (0)
	CD56	40 (7)	22 (4)	22 (4)	10 (2)	6 (1)
	CD25	94 (17)	6 (1)	0 (0)	0 (0)	0 (0)

*Percentage of cases (n-number of tissue samples).

**Table 3:** The immunoreactivity of CD3, CD56, CD69, CD25 antigens within decidua during the cesarean section.

Cesarean section	Cesarean section	Cesarean section with symptoms	Cesarean section with	P value
	without labor	of spontaneous beginning of labor	advanced labor	
CD3 (median ± SEM)	1 (±0.25)	3 (±0.32)	1 (±0.49)	.0257
CD69 (median ± SEM)	0 (±0.25)	1 (±0.35)	0 (±0.16)	.05
CD56 (median ± SEM)	0.5 (±0.28)	1 (±0.56)	2 (±0.42)	.47
CD25 (median ± SEM)	0 (±0)	0 (±0.12)	0 (±0)	.5

**Table 4:** Comparison between the number of CD3 positive and CD56 positive cells and the level of immunoreactivity of CD69, and CD25 antigens in case of emergency cesarean hysterectomies and cesarean section at term: control group.

Variables	Cesarean section at	Emergency cesarean	P value
	term—control group	hysterectomies	
CD3 (median ± SEM)	3 (±0.33)	0 (±0.65)	.03
CD69 (median ± SEM)	1 (±0.27)	0 (±0.32)	.55
CD56 (median ± SEM)	1 (±0.4)	0 (±0.12)	.03
CD25 (median ± SEM)	0 (±0.08)	0 (±0)	.4
